# Genome-wide analysis of the *GID* gene family in soybean and analysis of expression under gibberellin treatment

**DOI:** 10.3389/fpls.2026.1824314

**Published:** 2026-05-20

**Authors:** Zhiyuan Yao, Chenqiang Lv, Yuanyuan Zhang, Jingkun Yang, Shanshan Huang, Junqian Wang, Zhimin Hao, Chaoyue Zhao, He Sun, Wenqi Sun, Lu Liu, Hansheng Wang, Qi Zhang, Piwu Wang

**Affiliations:** Biotechnology Center, Jilin Agricultural University, Changchun, China

**Keywords:** expression quantity, GA treatment, gene family, GID, soybean

## Abstract

Soybean is a major grain and oil crop worldwide. Plant hormones play a critical role in regulating soybean growth and development. The *GID* gene family plays an important role in gibberellin (GA) perception and signaling, thereby contributing to the regulation of endogenous GA-mediated growth and developmental processes.signaling In this study, subcellular localization prediction indicated diverse potential localization patterns among the GmGID proteins, while experimental transient expression suggested that *GmGID5* may be associated with chloroplasts. Comprehensive analyses were conducted, including gene structure, conserved motifs, phylogenetic relationships, and expression patterns. Phylogenetic analysis classified these genes into six subgroups, revealing close evolutionary relationships with their homologs in *Arabidopsis*. These *GID* genes were distributed across 17 chromosomes. Synteny analysis demonstrated collinearity among soybean and other species, including rice, maize, and *Arabidopsis*. Expression profiling indicated that *GID* genes exhibit diverse expression patterns in different tissues, including the roots, stems, leaves, and seeds, with the majority of the genes showing high expression levels in the leaves. Quantitative reverse transcription PCR (qRT-PCR) analysis was performed on three representative genes: GmGID1, GmGID2, and GmGID3. The results showed that these genes exhibited altered expression patterns under GA treatments at concentrations of 30, 50, and 80 ppm, indicating their potential involvement in GA-mediated responses.signaling Subcellular localization analysis showed that *GmGID5* was localized in chloroplasts. Functional annotation and expression analyses suggested that soybean *GID* genes may be involved in GA signaling, reproductive development, and stress responses. In addition, eight miRNAs were predicted to regulate stress responses, modulate seed oil and protein metabolism, and coordinate with *GID* genes to influence soybean growth and stress adaptation. This study provides a theoretical foundation for further functional characterization of *GID* genes and offers valuable insights for the breeding of high-yield and stress-tolerant soybean varieties.

## 1 Introduction

Soybean is one of the most important crops worldwide, serving as a major source of plant-derived protein and oil (Rochaix and Ramundo, 2017). It is widely used in the food industry, ranging from traditional soy-based products to various edible oils, making it an indispensable agricultural commodity (Wu et al., 2024). In the feed industry, soybean meal is a high-quality protein source essential for livestock production (Herman and Schmidt, 2016). With the continued growth of the global population and the rising living standards, the demand for soybean and soybean-derived products is steadily increasing. Consequently, enhancing soybean yield and quality has become a central objective in agricultural research (Anderson et al., 2017). Plant growth and development are regulated by complex interactions between environmental factors and hormones. Among these, DELLA proteins act as key transcriptional regulators in hormone signaling pathways. These proteins are modulated by gibberellins (GAs), thereby influencing processes such as rhizobial symbiosis and nitrogen fixation efficiency.

GAs are essential plant hormones that regulate numerous developmental processes, including seed germination, stem elongation, leaf expansion, flowering, and fruit development (Ramon et al., 2020). The GA signaling pathway functions through hormone perception and downstream signal transduction events that ultimately regulate gene expression (Wang et al., 2020). The GID1 protein functions as the GA receptor and plays a central role in this pathway (Ueguchi-Tanaka et al., 2005). Upon GA binding, GID1 undergoes a conformational change that enhances its interaction with DELLA proteins, which act as negative regulators of GA signaling. The resulting GA–GID1–DELLA complex is recognized by the SCF complex, leading to ubiquitination and the subsequent degradation of DELLA proteins (Dill et al., 2001). The degradation of DELLA proteins relieves repression on the downstream growth-related genes, thereby activating GA-responsive pathways and promoting plant growth and development (Blanco-Touriñán et al., 2020).

*GID1* genes have been identified and functionally characterized in several plant species, demonstrating their conserved and diversified roles in GA signaling. In rice, *OsGID1* was identified as a soluble GA receptor, and mutations in *OsGID1* result in GA-insensitive dwarf phenotypes, confirming its essential role in GA perception and plant height regulation (Ueguchi-Tanaka et al., 2005). In *Arabidopsis*, three *GID1* homologs—*AtGID1A*, *AtGID1B*, and *AtGID1C*—have been reported. These receptors function redundantly, but also show functional divergence in regulating seed germination, stem elongation, fertility, and other GA-dependent developmental processes (Ueguchi-Tanaka et al., 2005; Griffiths et al., 2006). In wheat, *TaGID1* homologs have also been isolated and shown to share structural similarity with *OsGID1*, suggesting that GID1-mediated GA perception is conserved among cereal crops (Li et al., 2013). In addition, evolutionary studies have indicated that the *GID1* family has undergone expansion and functional diversification during land plant evolution, particularly in angiosperms (Yoshida et al., 2018).

As key components of the GA signaling pathway, *GID1* genes play central roles in regulating plant responses to GA (Xu et al., 2021). Upon GA perception, the receptor *GID1* binds to bioactive GA and forms a GA–GID1 complex, which subsequently interacts with DELLA proteins. This interaction facilitates the recruitment of F-box proteins, such as *GID2* in rice, leading to the ubiquitination and proteasomal degradation of DELLA proteins, thereby regulating plant growth and development. It should be noted that *GID1* genes encode GA receptors rather than members of the DELLA subfamily or the GRAS family. DELLA proteins, as a subfamily of GRAS proteins, function as negative regulators of GA signaling and occupy central regulatory roles in GA-mediated growth responses (Guo et al., 2017; To et al., 2020), whose members occupy central regulatory roles in the GA signaling pathway. GRAS proteins are generally involved in promoting the GA signaling pathway (Wu et al., 2023). However, in *GID* transgenic plants, GA signaling is enhanced, accompanied by reduced expression of RGA and GAI, which negatively regulate stem elongation. On the other hand, endogenous bioactive GAs, including GA4 and GA7, show significant increases. Studies on model plants, such as *Arabidopsis* and rice, have demonstrated that mutations in *GID* genes can alter plant sensitivity to GA, thereby affecting key agronomic traits, including plant height, internode elongation, and flowering (Yamaguchi et al., 2016). Although *GID1* genes have been studied in model plants and several crop species, systematic identification and characterization of the *GID* gene family in soybean remain limited. Soybean has experienced whole-genome duplication events, which may have contributed to the expansion and functional diversification of the gene families involved in hormone signaling. Therefore, the identification of soybean *GmGID* genes and analysis of their gene structures, evolutionary relationships, expression profiles, and potential responses to GA treatment are important for understanding their roles in soybean growth and development. Therefore, a comprehensive analysis of the composition, gene structure, expression patterns, and functions of the soybean *GmGID* gene family across different development stages and under stress conditions will not only enhance our understanding of the molecular mechanisms underlying soybean growth and development but also provide valuable genetic resources for soybean improvement (Du et al., 2023).

The objectives of this study were to identify members of the *GmGID* gene family, analyze their gene structures and evolutionary relationships, and investigate their expression patterns in different tissues. These findings provide a theoretical basis for further functional characterization of the *GmGID* genes in soybean growth and development. This study contributes to a deeper understanding of GA signaling regulation and offers potential gene targets for soybean genetic improvement, facilitating the development of high-yield and high-quality soybean varieties.

## 2 Materials and methods

### 2.1 Plant materials and stress treatments

The soybean cultivar JN136 was obtained from the Biotechnology Center of Jilin Agricultural University. Uniform seeds with full development were sown in soil and grown in a controlled culture chamber under the following conditions: 25°C with a 16-h light/8-h dark photoperiod. Soybean seedlings at the V2 stage were treated with GA at concentrations of 30, 50, and 80 ppm. These concentrations were selected based on previous studies using GA_3_ treatments in leguminous crops, where concentrations of 30, 50, 70, 90, and 110 ppm were applied and shown to affect plant growth parameters (Noor, 2017).

### 2.2 Identification of the *GmGID* gene family

The gene sequences, gene annotations, and amino acid sequences were retrieved from the Phytozome 13 plant genome database (https://phytozome-next.jgi.doe.gov/) (the genome database named soybean Wm82.a2.v1). The hidden Markov model (HMM) of the *GID* gene family (PF07859; https://www.ebi.ac.uk/interpro/entry/pfam/PF07859/) was obtained from the Pfam database (https://www.ebi.ac.uk/interpro/) (Mistry et al., 2020). Candidate GID proteins in the soybean genome were identified using HMMER3.0 (http://hmmer.org/) with default parameters. A BLASTp search was performed to complement the HMM-based screening. An *E*-value threshold of 1 × 10^−5^ was applied in both HMMER and BLASTp analyses, and the results from the two methods were integrated to obtain a non-redundant set of *GID* gene family members. The physicochemical properties of the members of the *GmGID* family, including the molecular weight (MW), the isoelectric point (p*I*), and the amino acid length, were predicted using the ExPASy ProtParam online tool (https://web.expasy.org/protparam/). Subcellular localization of the proteins was predicted using Cell-Ploc2.0 (http://www.csbio.sjtu.edu.cn/bioinf/plant/) (Chen et al., 2020).

### 2.3 Construction of the phylogenetic tree of the *GmGIDs* genes

Multiple sequence alignment of the GID protein sequences of soybean, rice, maize, and *Arabidopsis* was performed using ClustalW with default parameters (Hung and Weng, 2016). Based on the aligned sequences, an unrooted phylogenetic tree was constructed using the neighbor-joining (NJ) method implemented in MEGA11 (Kumar et al., 2018). Bootstrap analysis was conducted with 1,000 replicates. The resulting tree was exported in Newick (NWK) format and subsequently visualized and refined using the ChiPlot online platform (https://www.chiplot.online/).

### 2.4 Chromosome mapping and collinearity analysis of the *GmGIDs*

For chromosomal localization, the physical positions of the *GmGID* genes were extracted from the soybean genome annotation file, and their distribution across chromosomes was visualized using TBtools (Chen et al., 2020).

For interspecific collinearity analysis, protein sequence alignments between soybean and rice, as well as between soybean and *Arabidopsis*, were performed using BLASTP (https://blast.ncbi.nlm.nih.gov/) (Chen et al., 2020). The top 5 alignment hits were retained and the redundant matches removed (Camacho et al., 2009). Collinear relationships between species were visualized using Circos (http://circos.ca/) (Lohani et al., 2021). In addition, the collinearity block information generated by MCScanX was converted into a Circos-compatible input format (Krzywinski et al., 2009). The MCScanX output files were directly imported into the “AdvancedCircos” module of TBtools to generate collinearity plots (Wang et al., 2012).

For intraspecific collinearity analysis, a BLASTP search was performed on the soybean proteome with an *E*-value cutoff of 1 × 10^−10^ to identify reliable homologous gene pairs (https://blast.ncbi.nlm.nih.gov/) (Zhang et al., 2016). The top 5 hits were retained and the redundant alignments removed (Li et al., 2023). The alignment results were in BLASTP standard format (Yu et al., 2025). Intraspecific collinearity analysis was performed using MCScanX software (The UniProt Consortium, 2015). The resulting BLASTP output, together with the gene annotation files containing the gene location information, was used as the input for MCScanX to detect collinear blocks based on gene order and genomic distribution (Wang et al., 2024, Wang et al., 2013). Identified collinearity relationships were then visualized using the “AdvancedCircos” function in TBtools (Zhang and Sun, 2009).

### 2.5 Analysis of the physicochemical properties of the *GmGID* gene family

The physicochemical properties of the proteins encoded by the *GmGID* gene family members were analyzed using the ExPASy ProtParam tool (El-Gebali et al., 2018). Parameters including amino acid composition, MW, theoretical isoelectric point (p*I*), protein half-life, and instability index were calculated. This analysis provided the relative proportions of the 20 common amino acids in each protein, as well as the theoretical MW and p*I* values, which are useful for predicting the protein stability and charge characteristics under different environments (Duvaud et al., 2021).

### 2.6 MOTIF, gene structure, and promoter analysis of the *GmGID* gene family

Conserved motifs in GmGID proteins were identified using the MEME online tool, with the maximum number of motifs set to 10 and all other parameters set to default values (Hu et al., 2014). Gene structure information, including exon–intron organization, conserved domains, and motif distribution, was integrated and visualized using TBtools-II (Bailey et al., 2015).

The 2-kb upstream sequences from the transcription start site of each *GmGID* gene were extracted using TBtools-II. These sequences were submitted to the PlantCARE database for the prediction of the *cis*-acting elements. The identified elements were categorized into four functional groups: “hormone,” “light response,” “environmental stress,” and “growth and development.” The distribution of these elements was visualized using TBtools-II.

### 2.7 Expression profiling of the *GmGID* genes in different soybean tissues

The expression profiles of the *GmGID* gene family members were obtained using the Plant Public RNA-seq database (PPRD; https://plantrnadb.com/) (Goodstein et al., 2011). The expression levels were analyzed across five tissues: root, stem, main stem, leaf, and seed. The expression data were visualized as heatmaps using the ChiPlot online tool (https://chiplot.online/circle_heatmap.html) and further processed and plotted using TBtools-II software.

### 2.8 Expression of *GmGID1–3* genes after GA treatment

Soybean seedlings at the three-leaf stage (7–10 days after sowing) were used for the GA treatment experiments. Four treatment groups were established: 0 ppm (water control) and 30, 50, and 80 ppm GA, representing low-, medium-, and high-concentration treatments, respectively. At 9:00 a.m. on the day of treatment, seedlings were uniformly sprayed with the corresponding GA solutions (10 ml per plant) under low-light conditions. Samples were collected at 0.5, 2, and 6 h after treatment to assess early transcriptional responses.

Based on the conserved domain integrity and the motif composition, three representative genes—*GmGID1*, *GmGID2*, and *GmGID3*—were selected for quantitative reverse transcription PCR (qRT-PCR) validation. Total RNA was extracted from the collected tissues and reverse-transcribed into first-strand complementary DNA (cDNA). qRT-PCR was performed using gene-specific primers, with *β-actin* serving as the internal control. The relative gene expression levels were calculated using the 2^−ΔΔCt^ method. Each treatment included three biological replicates, with three technical replicates per biological sample.

Amplification was conducted on an Mx3000P Real-Time PCR System (Agilent Technologies, Lexington, MA, USA). Relative expression data are presented as the mean ± standard deviation (SD). Statistical significance among the different GA treatment groups was determined using one-way analysis of variance (ANOVA) followed by Tukey’s multiple comparison test, with a significance threshold of p < 0.05. Data are presented as the mean ± SD from three biological replicates.

### 2.9 Subcellular localization analysis of *GmGID5*

*GmGID5* was selected for subcellular localization analysis as a representative member of the *GmGID* family due to its intact conserved domain and strong bioinformatics prediction of chloroplast localization.

For transient expression analysis, the full-length coding sequence of *GmGID5*, excluding the stop codon, was amplified and fused in-frame to the green fluorescent protein (GFP) in the pCAMBIA1302 vector, generating the pCAMBIA1302–*GmGID5*–GFP construct. The recombinant plasmid was introduced into *Agrobacterium tumefaciens* GV3101. Leaves of 6- to 8-week-old tobacco plants were used for *Agrobacterium*-mediated transient transformation. The bacterial suspension carrying the fusion construct was infiltrated into the abaxial surface of tobacco leaves, while the empty pCAMBIA1302–GFP vector served as a control.

After 48 h of incubation, fluorescence signals were observed using a confocal laser scanning microscope. The GFP signals, chloroplast autofluorescence, and bright-field images were captured. The merged images were subsequently analyzed to determine the subcellular localization of *GmGID5*.

### 2.10 GO enrichment analysis and microRNA analysis of the *GmGID* gene family

All 54 identified *GmGID* genes were subjected to Gene Ontology (GO) enrichment analysis using gprofiler (https://biit.cs.ut.ee/gprofiler/gos) to predict their potential biological functions. MicroRNA (miRNA) analysis of the *GmGID* family members was performed using the psRNATarget online tool. The regulatory network between miRNAs and their corresponding target genes was subsequently visualized using the website http://www.bioinformatics.com.cn/plot_basic_miRNA_target_network_plot_197.

## 3 Results

### 3.1 Identification and phylogenetic analysis of the *GmGID* gene family

**Figure 1:**
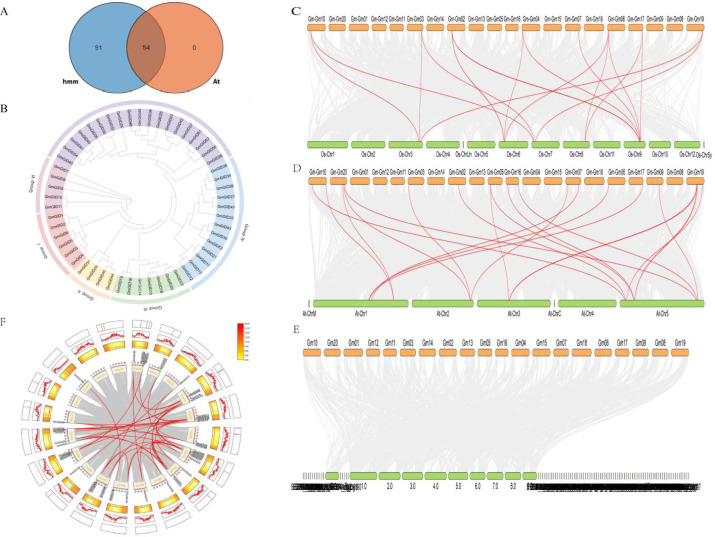
**(A)** Venn map of the GID family members in soybean. **(B)** Phylogenetic tree. **(C)** Collinearity analysis of the *GmGID* gene family in soybean. (**D**–**F**) Collinearity analyses of the *GID* gene family in soybean and *Arabidopsis* (**D**), in soybean and rice (**E**), and in soybean and corn (**F**). *HMM* represents candidate genes identified using the Pfam HMM profile of the *GID* family, while *At* denotes candidates obtained by BLASTP using the GID/GID1 protein sequences from *Arabidopsis* as queries. The overlapping gene set was subsequently validated through conserved domain analysis.

A total of 54 *GmGID* genes were identified in soybean through a combined approach using BLASTP and HMMER (**Figure 1A**). All candidate proteins were confirmed to contain complete conserved domains of the *GID* family, as verified by SMART and CDD. A phylogenetic tree of the *GmGID* gene family was subsequently constructed (**Figure 1B**). Detailed information on the 54 identified *GmGID* genes is provided in **Table 1**. Analysis of the physicochemical properties revealed considerable variation among family members. *GmGID48* encoded the smallest protein with 289 amino acids (aa), whereas *GmGID44* and *GmGID45* encoded the largest proteins with 451 aa. The predicted p*I* ranged from 5.14 (*GmGID14*) to 8.97 (*GmGID10*). Subcellular localization predictions indicated that many of the GmGID proteins are localized to the chloroplasts and the cytoskeleton. *GmGID17*, *GmGID21*, and *GmGID38* were predicted to localize exclusively to the mitochondria, *GmGID18* to the nucleus, and *GmGID14* to the peroxisome.

**Table 1:** Analysis results of the protein physicochemical properties.

Gene	Gene ID	Length (aa)	MolWt	p*I*	Subcellular localization
*Glyma.10G158000.1.p*	*GmGID1*	344	38,891.29	6.47	Cytoplasmic vesicles and cytoskeleton
*Glyma.20G230600.2.p*	*GmGID2*	344	38,669.04	5.95	Cytoplasmic vesicles and cytoskeleton
*Glyma.02G151100.1.p*	*GmGID3*	342	38,790.93	6.02	Cytoplasmic vesicles and cytoskeleton
*Glyma.10G022900.1.p*	*GmGID4*	343	38,986.34	6.52	Cytoplasmic vesicles and cytoskeleton
*Glyma.03G148300.1.p*	*GmGID5*	346	39,391.72	7.16	Chloroplast
*Glyma.19G151601.1.p*	*GmGID6*	346	39,208.54	7.11	Chloroplast
*Glyma.16G208100.1.p*	*GmGID7*	338	37,794.81	7.68	Chloroplast
*Glyma.09G157600.2.p*	*GmGID8*	337	37,466.62	8.92	Chloroplast
*Glyma.16G208200.1.p*	*GmGID9*	338	37,397.42	6.32	Chloroplast
*Glyma.09G157700.1.p*	*GmGID10*	361	40,591.35	8.97	Chloroplast
*Glyma.16G208300.1.p*	*GmGID11*	331	37,324.26	6.18	Chloroplast
*Glyma.18G298000.1.p*	*GmGID12*	340	3,7816.41	8.58	Chloroplast
*Glyma.08G364100.1.p*	*GmGID13*	362	40,480.24	8.8	Chloroplast
*Glyma.06G308500.1.p*	*GmGID14*	329	36,714.51	5.14	Peroxisome
*Glyma.03G205400.1.p*	*GmGID15*	324	36,445.66	5.95	Cytoplasmic vesicles and cytoskeleton
*Glyma.13G189800.3.p*	*GmGID16*	382	43,214.78	5.97	Chloroplast
*Glyma.20G113800.1.p*	*GmGID17*	352	39,095.39	9.21	Mitochondrion
*Glyma.19G202900.1.p*	*GmGID18*	324	36,511.7	6.32	Nuclear
*Glyma.06G310100.1.p*	*GmGID19*	338	37,734.09	5.53	Cytoplasmic vesicles and cytoskeleton
*Glyma.10G087500.1.p*	*GmGID20*	332	37,467.59	6.05	Cytoplasmic vesicles and cytoskeleton
*Glyma.10G275900.2.p*	*GmGID21*	295	32,250.93	7.62	Mitochondrion
*Glyma.02G166800.1.p*	*GmGID22*	332	37,391.52	6.24	Cytoplasmic vesicles and cytoskeleton
*Glyma.01G239500.1.p*	*GmGID23*	320	35,198.12	5.82	Cytoplasmic vesicles and cytoskeleton
*Glyma.17G243700.1.p*	*GmGID24*	337	37,376.23	5.65	Cytoplasmic vesicles and cytoskeleton
*Glyma.01G239300.1.p*	*GmGID25*	333	36,844.93	6.56	Cytoplasmic vesicles and cytoskeleton
*Glyma.17G210000.1.p*	*GmGID26*	309	34,961.1	6.09	Cytoplasmic vesicles and cytoskeleton
*Glyma.09G149400.1.p*	*GmGID27*	324	36,637.8	4.98	Cytoplasmic vesicles and cytoskeleton
*Glyma.20G153200.1.p*	*GmGID28*	337	37,324.01	8.63	Chloroplast
*Glyma.19G078200.3.p*	*GmGID29*	441	48,562.4	8.31	Chloroplast
*Glyma.04G037400.1.p*	*GmGID30*	315	35,088.52	5.06	Cytoplasmic vesicles and cytoskeleton
*Glyma.05G073200.1.p*	*GmGID31*	435	48,052.17	8.79	Chloroplast
*Glyma.06G038000.1.p*	*GmGID32*	326	36,356.94	5.34	Cytoplasmic vesicles and cytoskeleton
*Glyma.20G153300.1.p*	*GmGID33*	329	36,542.54	5.88	Cytoplasmic vesicles and cytoskeleton
*Glyma.16G201000.2.p*	*GmGID34*	372	41,534.64	8.23	Chloroplast
*Glyma.02G134000.1.p*	*GmGID35*	333	36,872.82	5.76	Cytoplasmic vesicles and cytoskeleton
*Glyma.10G250300.1.p*	*GmGID36*	343	37,912.09	5.23	Chloroplast
*Glyma.10G250200.1.p*	*GmGID37*	331	36,683.66	5.52	Cytoplasmic vesicles and cytoskeleton
*Glyma.09G149601.1.p*	*GmGID38*	354	39,549.33	8.84	Mitochondrion
*Glyma.07G082400.1.p*	*GmGID39*	319	35,607.77	6.59	Cytoplasmic vesicles and cytoskeleton
*Glyma.02G133800.1.p*	*GmGID40*	393	43,407.58	6.07	Chloroplast
*Glyma.02G133900.2.p*	*GmGID41*	322	35,499.19	5.11	Cytoplasmic vesicles and cytoskeleton
*Glyma.20G143400.1.p*	*GmGID42*	323	35,768.63	5.41	Cytoplasmic vesicles and cytoskeleton
*Glyma.03G020500.1.p*	*GmGID43*	319	35,878.09	6.79	Cytoplasmic vesicles and cytoskeleton
*Glyma.19G083100.1.p*	*GmGID44*	451	49,355.39	8.34	Chloroplast
*Glyma.16G062800.1.p*	*GmGID45*	451	49,419.59	8.53	Chloroplast
*Glyma.01G239600.1.p*	*GmGID46*	319	35,138.09	5.75	Cytoplasmic vesicles and cytoskeleton
*Glyma.16G200900.1.p*	*GmGID47*	318	35,228.31	5.44	Chloroplast
*Glyma.11G004200.1.p*	*GmGID48*	289	31,790.24	6.03	Cytoplasmic vesicles and cytoskeleton
*Glyma.07G211100.3.p*	*GmGID49*	384	42,527.65	6.16	Cytoplasmic vesicles and cytoskeleton
*Glyma.02G134100.1.p*	*GmGID50*	302	33,560.19	6	Chloroplast
*Glyma.07G211000.1.p*	*GmGID51*	304	33,533.1	5.59	Cytoplasmic vesicles and cytoskeleton
*Glyma.02G134200.1.p*	*GmGID52*	304	33,694.25	5.79	Cytoplasmic vesicles and cytoskeleton
*Glyma.07G082500.1.p*	*GmGID53*	334	36,373.5	5.65	Nuclear
*Glyma.07G211200.1.p*	*GmGID54*	308	34,695.83	5.96	Cytoplasmic vesicles and cytoskeleton

*MolWt*, molecular weight; *aa*, amino acids.

### 3.2 Collinearity analysis of the *GmGID* gene family

To further investigate the evolutionary relationships of the *GmGID* gene family, interspecific collinearity analysis was performed between soybean and *Arabidopsis*, rice, and maize. A total of 19 and 16 collinear gene pairs were identified between soybean and *Arabidopsis* and between soybean and rice, respectively, whereas no collinear gene pairs were detected between soybean and maize (**Figures 1C–E**). Soybean chromosome 19 exhibited the highest number of collinear relationships, with up to four homologous gene pairs. In addition, *GmGID* genes displayed syntenic relationships with *Arabidopsis* chromosomes 1, 2, 3, and 5. The abundance of *GID* homologous gene pairs indicated a high degree of evolutionary conservation of the *GID* gene family between soybean and *Arabidopsis*.

### 3.3 Intraspecific collinearity analysis

Gene duplication events, along with the retention of duplicated sequences, are key drivers of plant genome expansion and functional diversification. As shown in **Figure 1C**, a total of 23 duplication events were identified across 20 chromosomes of soybean. Among these chromosomes (designated Gm1–Gm20), the 54 *GmGID* genes were unevenly distributed, being present on 17 chromosomes, while no *GmGID* genes were detected on chromosomes Gm12, Gm14, and Gm15. In the Circos plot (**Figure 1F**), 23 segmentally duplicated gene pairs were identified and connected by red lines. These results suggest that segmental duplication was an important contributor to the expansion of the *GmGID* gene family. Tandem duplication may also have contributed to local gene clustering on specific chromosomes; however, its relative contribution requires further classification of the duplication types. Taken together, these results suggest that both tandem and segmental duplication events contributed to the expansion of the *GmGID* gene family.

### 3.4 Chromosome mapping of the *GmGID* gene family, MOTIF, and gene structure analysis

**Figure 2:**
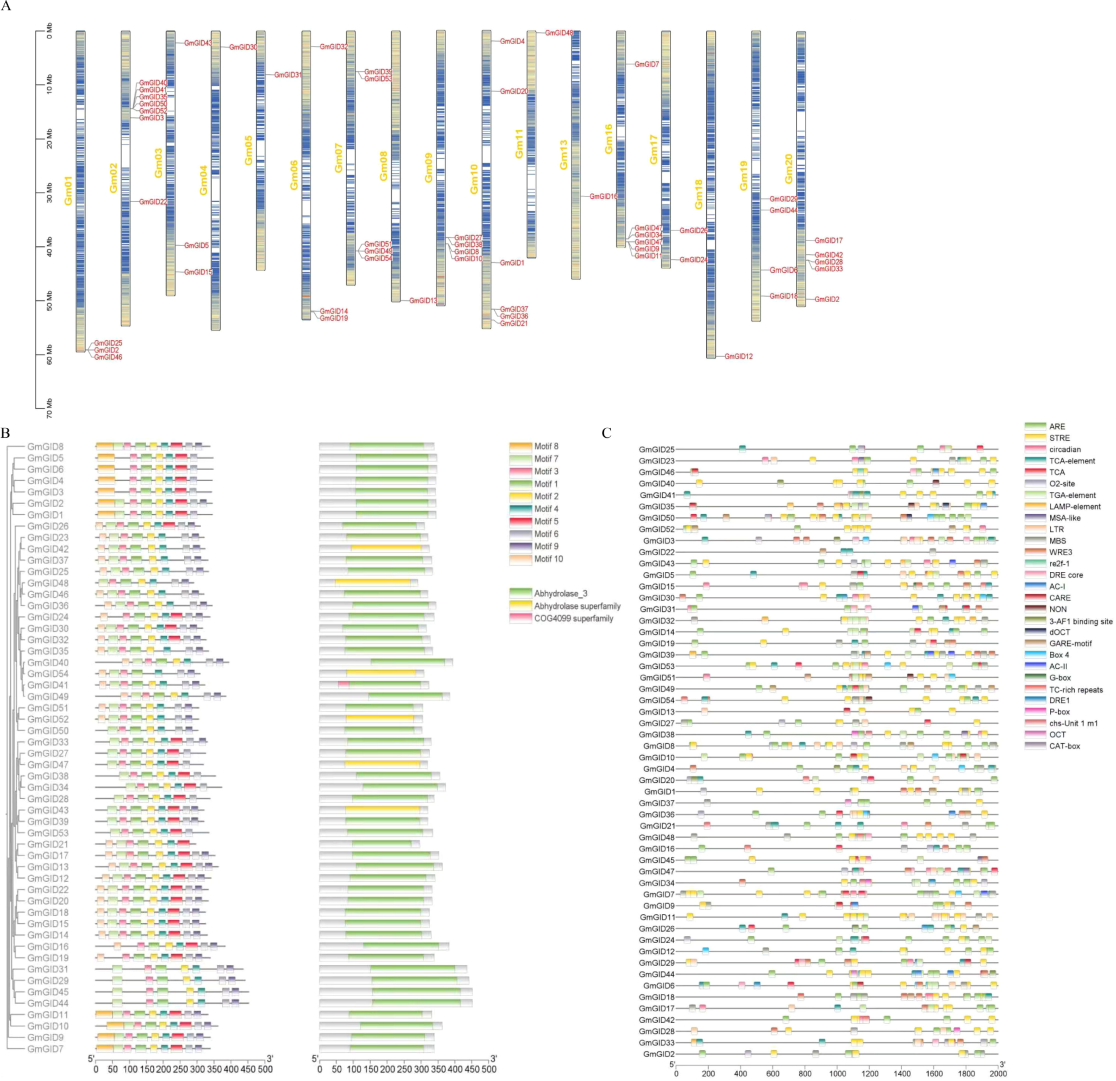
Chromosomal distribution, MOTIF, and gene structure of the *cis*-acting element analysis of the promoter regions of the *GmGID* gene family. **(A)** Gene structure. **(B)** Analysis of the *cis*-acting element. **(C)** Chromosomal distribution.

The 54 identified *GmGID* genes were unevenly distributed across 17 soybean chromosomes (**Figure 2A**). Among them, chromosomes Gm02, Gm07, Gm09, Gm10, Gm16, and Gm20 contain relatively higher numbers of *GmGID* genes. Gene structure analysis revealed that all *GmGID* genes contain multiple exons and introns (**Figure 2A**). Notably, genes that clustered within the same phylogenetic tree exhibited comparable numbers and lengths of exons and introns. All *GmGID* genes possess conserved GID-associated domains, representing signature features of this gene family (**Figure 2B**). Motif analysis using the MEME tool identified 10 conserved motifs (Motif 1–Motif 10) across the GmGID proteins. All *GmGID* genes exhibited a largely conserved motif composition, while closely related members displayed similar sequence features and motif arrangements. The presence of shared conserved motifs among the *GmGID* family members suggests functional similarity and further supports the reliability of the inferred phylogenetic relationships.

### 3.5 Analysis of the promoter *cis*-acting elements of the *GmGID* gene family

Given that gene expression is largely regulated by upstream promoter regions, the *cis*-acting elements within the 2,000-bp upstream promoter sequences of the 54 identified *GmGID* genes were systematically analyzed (**Figure 2C**). The results demonstrated that all *GmGID* genes contain multiple *cis*-elements associated with abiotic and biotic stress responses, phytohormone signaling, and plant growth and developmental processes. Among them, stress-responsive elements were the most abundant, whereas elements related to hormone signaling and growth/development were present at comparable levels. Notably, several well-characterized *cis*-elements, including MYB, ABRE, and P-box, are widely distributed across the promoters. In addition, a small number of MBS, CAT-BOX, and TC-rich repeats were also identified. Therefore, the *GmGID* gene family may participate in the responses to hormones such as GA and abscisic acid (ABA). Overall, these findings indicate that the *GmGID* gene family is potentially involved in hormone signaling, abiotic stress responses, and plant growth and developmental regulation.

### 3.6 Expression profiling of the *GmGID* genes across different tissues of soybean

**Figure 3:**
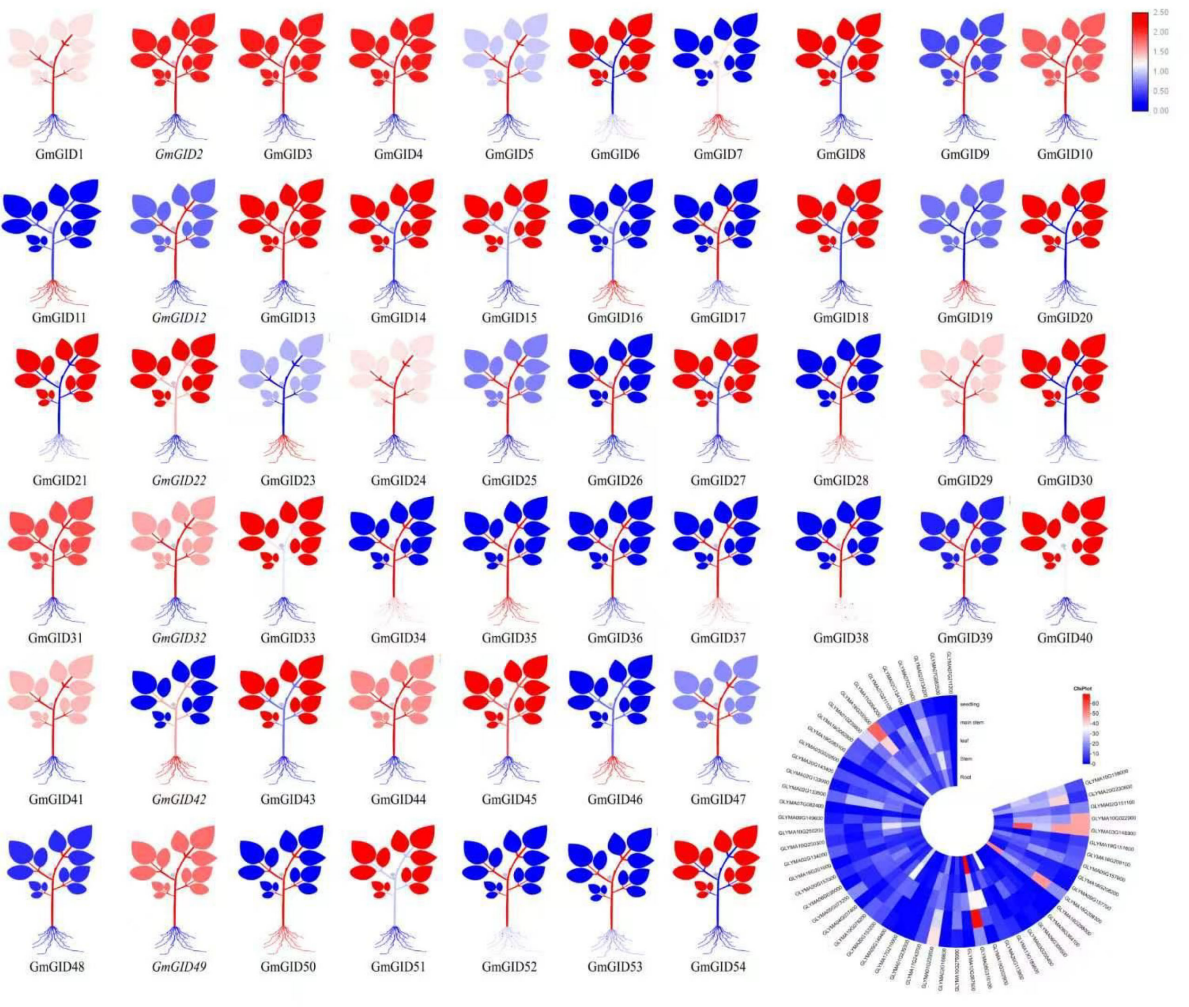
Expression profiling of the *GmGID* gene family in different soybean tissues.

Tissue-specific expression data of the *GmGID* genes were obtained from PPRD and visualized as tissue expression maps and a heatmap summary (**Figure 3**). As these visualizations were generated using different normalization methods and display scales, their color intensities are not directly comparable. Overall, the *GmGID* genes exhibited diverse tissue-specific expression patterns across roots, stems, leaves, and seeds. Several genes, including *GmGID7*, *GmGID11*, *GmGID16*, *GmGID19*, *GmGID23*, *GmGID28*, *GmGID35*, *GmGID42*, *GmGID46*, and *GmGID50*, showed elevated transcript abundance in the roots, suggesting potential roles in root growth and development. Many genes were also highly expressed in the leaves and stems, indicating potential involvement in leaf development and photosynthesis-related processes. The majority of the *GmGID* genes were expressed in at least one tissue, suggesting broad functional roles in soybean growth and development.

### 3.7 Analysis of the expression levels under GA treatment

**Figure 4:**
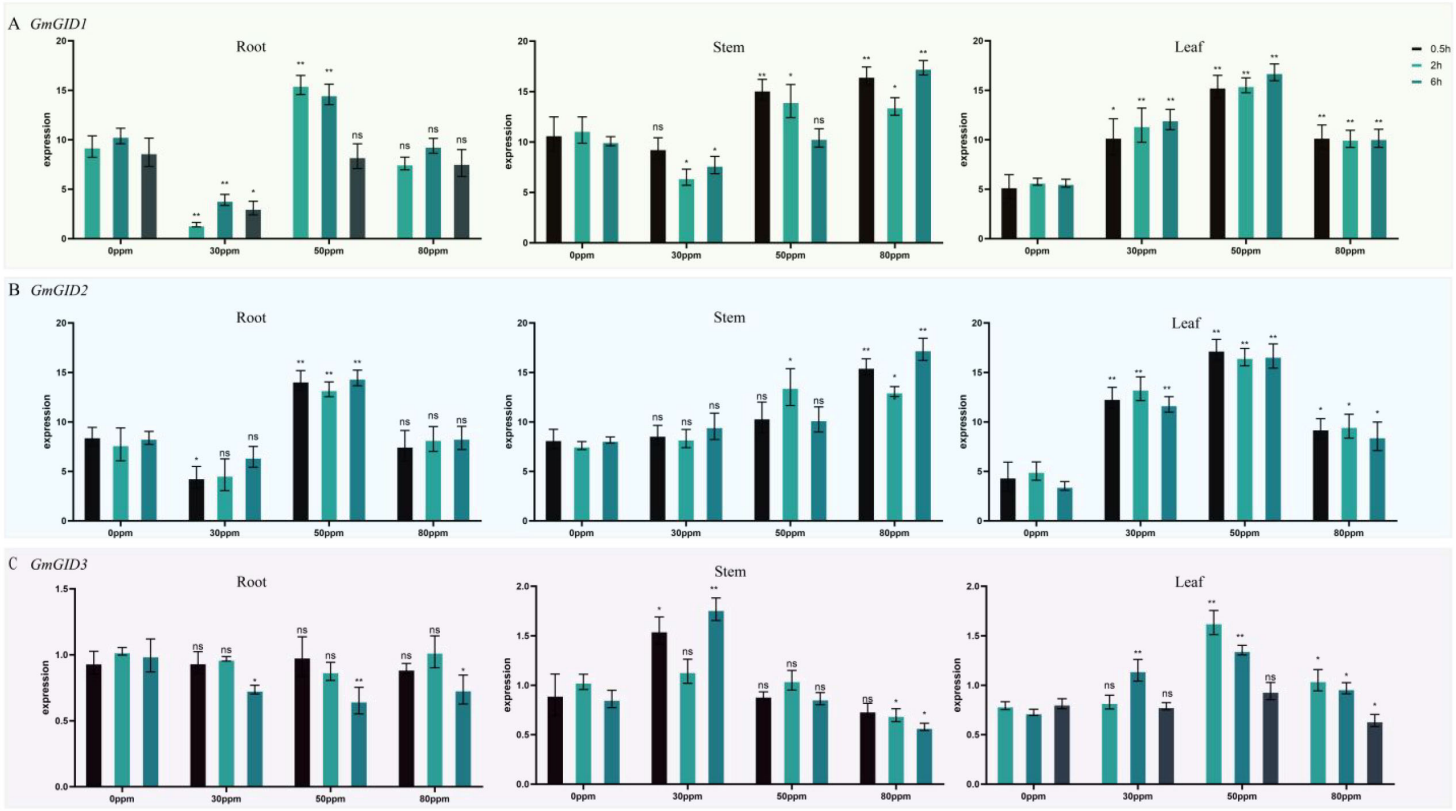
Expression analysis of the *GmGID* genes under gibberellin (GA) treatment. **(A–C)** Expression patterns of *GmGID1* **(A)**, *GmGID2* **(B)**, and *GmGID3* **(C)** under different GA concentrations. Soybean seedlings were treated with 0, 30, 50, and 80 ppm GA, and samples were collected at 0.5, 2, and 6 h after treatment. Expression levels were analyzed in the roots, stems, and leaves using quantitative reverse transcription PCR (qRT-PCR). Data are presented as the mean ± standard deviation (SD) from three biological replicates. Statistical significance among the different GA treatment groups was determined using one-way analysis of variance (ANOVA) followed by Tukey’s multiple comparison test. *Different letters* indicate significant differences among treatment groups at *p* < 0.05. ns, no significant difference; *P < 0.05; **P < 0.01.

5/5000 As shown in **Figure 4**, *GmGID2* showed tissue- and concentration-dependent responses to GA treatment. Its expression was reduced under 30 ppm GA, but was markedly increased under 50 ppm in the roots. It gradually increased with higher GA concentrations in the stems and was strongly induced under 30 and 50 ppm in the leaves. However, expression in the leaf decreased under 80 ppm compared with 50 ppm. These results indicate that *GmGID2* responded to GA treatment in a tissue-dependent manner, with stronger induction under specific GA concentrations.

Overall, *GmGID1*, *GmGID2*, and *GmGID3* exhibited divergent GA-responsive expression patterns rather than a uniform trend. Their expression levels varied according to the GA concentration, the treatment duration, and the tissue type. Among the three genes, *GmGID1* and *GmGID2* showed stronger transcriptional responses than *GmGID3*, suggesting that they may play more active roles in GA-mediated transcriptional regulation. 

### 3.8 Subcellular localization analysis of *GmGID5*

**Figure 5:**
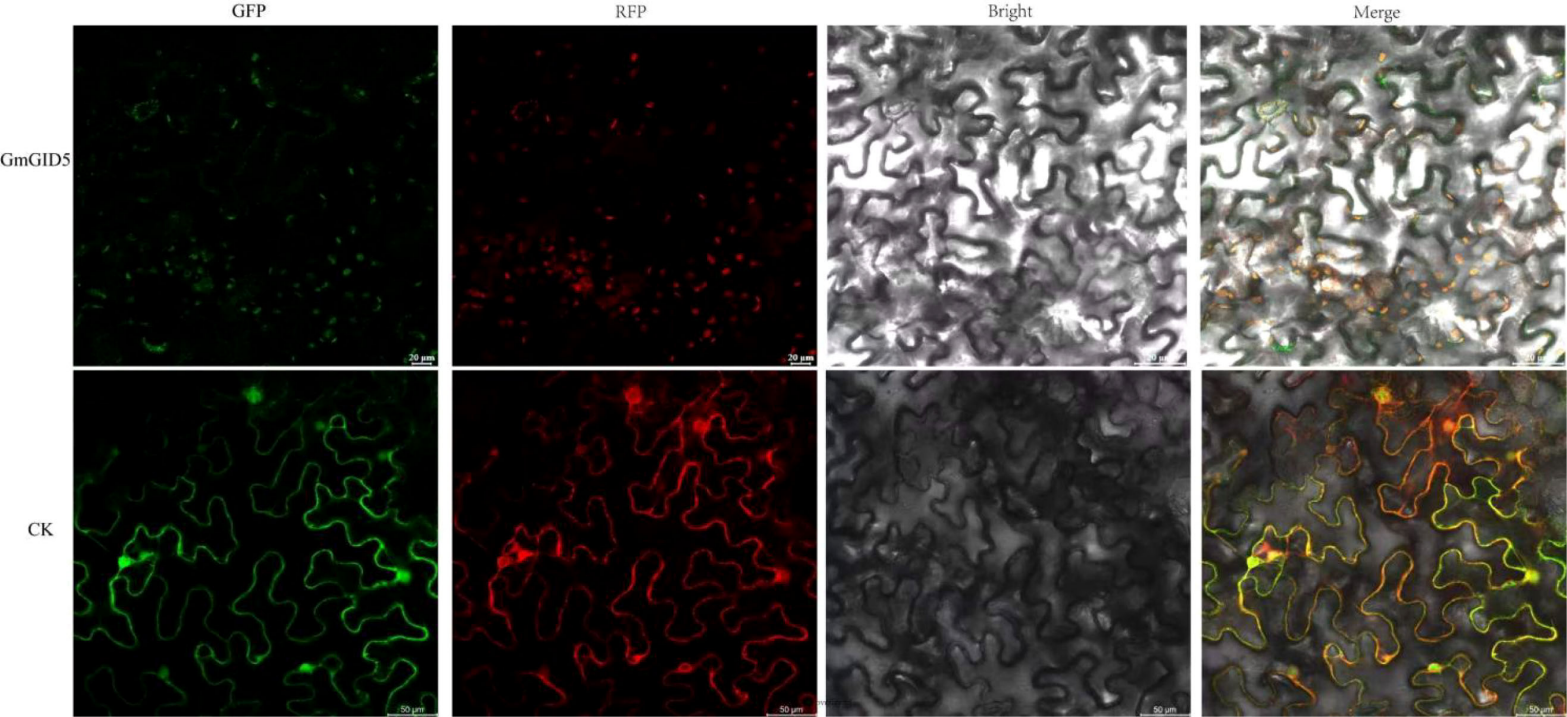
Subcellular localization results (CK bars = 50 μm, *GmGID5* bars = 20 μm). *GFP* green fluorescent protein; *RFP* red fluorescent protein. *Bright* signifies bright field and *Merge* indicates overlay field.

To examine the predicted subcellular localization of *GmGID5*, the pCAMBIA1302–*GmGID5*–GFP fusion construct was transiently expressed in tobacco leaf epidermal cells. As shown in **Figure 5**, the GFP fluorescence signal of *GmGID5* overlapped with chloroplast autofluorescence, suggesting a possible chloroplast-associated localization of *GmGID5*. However, because chloroplast autofluorescence was used as the reference signal, this localization pattern should be further confirmed using organelle-specific markers or stable transformation systems.

### 3.9 GO enrichment analysis and miRNA analysis of the *GmGID* gene family

**Figure 6:**
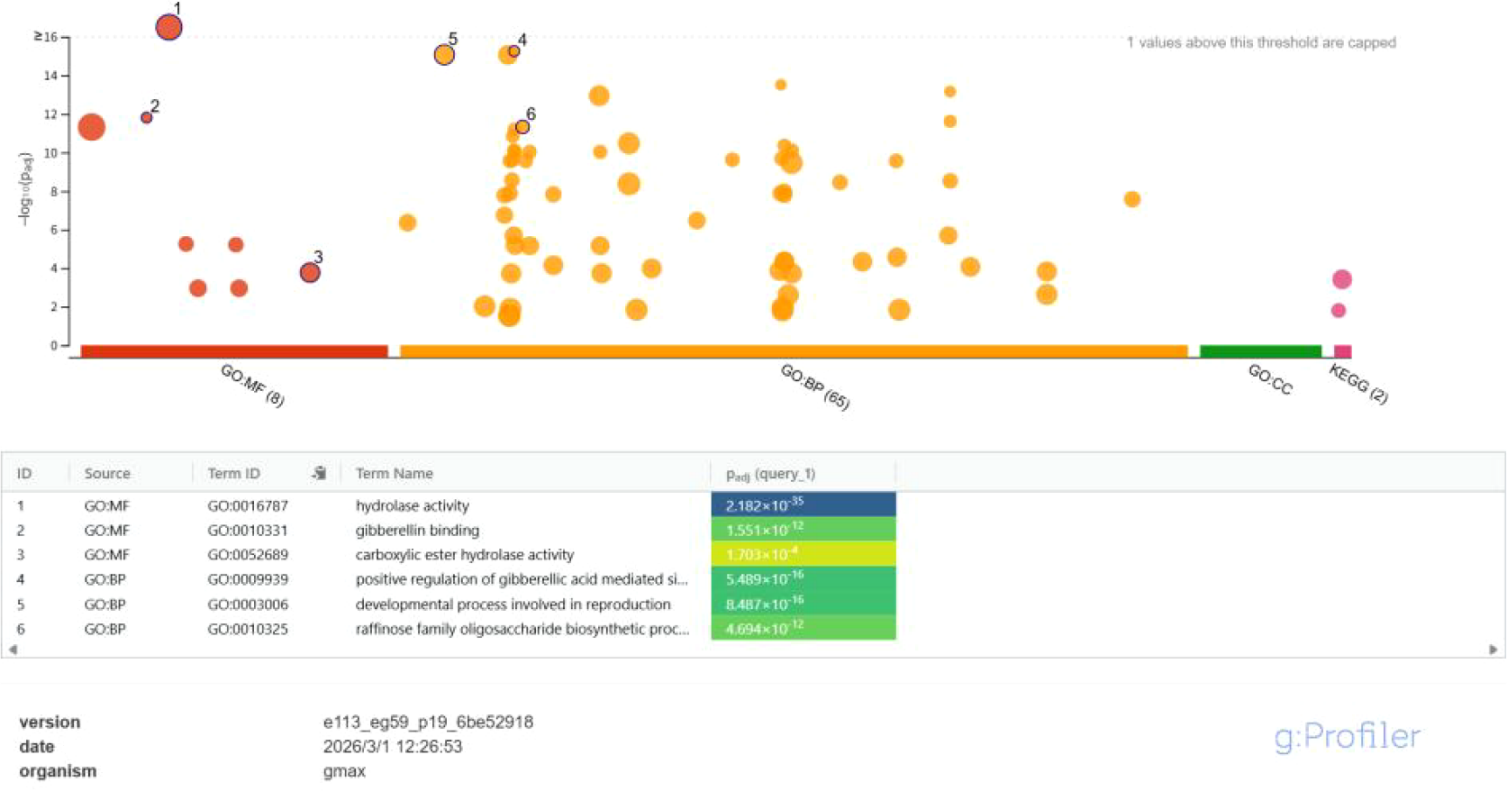
Gene Ontology (GO) enrichment analysis of the *GmGID* gene family.

GO enrichment analysis was performed to predict the potential functional categories associated with the *GmGID* gene family. The results suggest that the *GmGID* genes may be associated with functional entries related to phytohormone regulation, reproductive development, and secondary metabolism (**Figure 6**). At the molecular function (GO: MF) level, hydrolase activity was the most significantly enriched term, followed by GA binding and carboxylate hydrolase activity, suggesting that GmGID proteins may be related to enzymatic processes and hormone signal perception at the prediction level. At the biological process (GO: BP) level, enriched terms included positive regulation of GA-mediated signaling pathways and reproductive development, indicating that *GmGID* genes may be potentially involved in hormone-mediated regulation and developmental processes. Overall, these GO enrichment results provide preliminary clues for the possible roles of *GmGID* genes in GA signaling, reproductive development, and oligosaccharide metabolism; however, these associations require further experimental validation.

**Figure 7:**
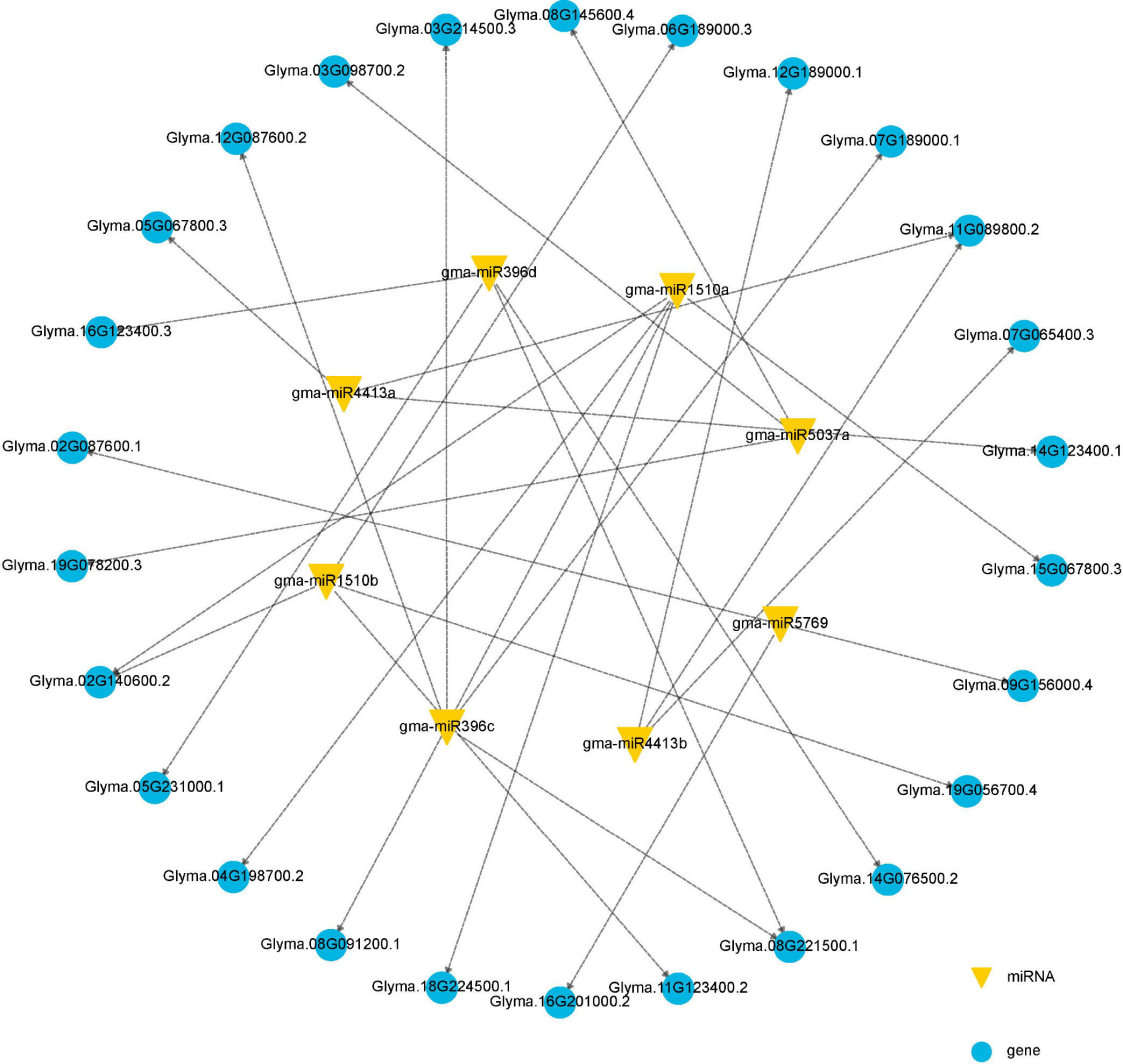
Network diagram of eight miRNAs and their corresponding target genes in the *GmGID* gene family.

MicroRNA target prediction analysis identified eight candidate miRNAs potentially associated with *GmGID* genes (**Figure 7**). Functional annotation of the predicted target genes suggested possible involvement in biotic stress responses, growth and development regulation, abiotic stress adaptation, and seed quality formation in soybean. Among them, gma-miR1510a/b was predicted to target *NBS*-*LRR* disease resistance genes and other disease-related signaling components, suggesting a possible association with defense-related regulation. gma-miR396c/d was predicted to target *GRF* genes and drought-responsive genes, implying a potential link with soybean growth and stress adaptation. In addition, gma-miR5769, gma-miR5037a, and gma-miR4413a/b were predicted to be associated with seed oil biosynthesis, salt and low-temperature stress responses, and seed protein metabolism, respectively. These predicted miRNA–target relationships should be regarded as preliminary hypotheses and require experimental validation. 

## 4 Discussion

In this study, 54 *GmGID* genes were identified in soybean and classified into six subgroups. The conserved exon–intron structures and motif compositions within the same phylogenetic clades suggest that many *GmGID* members have retained structural conservation after duplication. Compared with model plants such as *Arabidopsis* and rice, the relatively large number of *GmGID* genes suggests an expansion of this family in soybean, providing a basis for functional diversification in GA-related growth regulation (Ueguchi-Tanaka et al., 2005; Griffiths et al., 2006; Gazara et al., 2018).

The expansion of the *GmGID* family may be largely associated with soybean paleopolyploidy and the retention of duplicated chromosomal segments. The detected duplicated gene pairs and syntenic relationships indicate that segmental duplication contributed to *GmGID* expansion, while the closer collinearity between soybean and *Arabidopsis* than with rice or maize is consistent with their shared dicot ancestry. After duplication, some *GmGID* paralogs may have retained conserved motifs, whereas others may have diverged in expression patterns or localization, suggesting possible sub-functionalization (Gazara et al., 2018; Du et al., 2023).

The diverse tissue expression patterns of the *GmGID* genes indicate possible functional divergence among family members. Genes with relatively high expression in the roots may be associated with root development or legume-specific symbiotic processes, whereas genes preferentially expressed in the leaves and stems may participate in GA-regulated vegetative growth. This interpretation is consistent with previous findings that GA/DELLA signaling participates in rhizobial infection and nodule organogenesis in legumes (Fonouni-Farde et al., 2016; Wang et al., 2020b).

qRT-PCR analysis further showed that *GmGID1*, *GmGID2*, and *GmGID3* responded differently to GA treatment depending on the tissue type, concentration, and treatment duration. These gene-specific responses may reflect different requirements for GA perception and DELLA-mediated transcriptional regulation in soybean tissues. This interpretation is consistent with the canonical GA–*GID1–*DELLA signaling model, in which *GID1* perceives bioactive GA and promotes DELLA degradation to regulate downstream GA-responsive growth processes (Ueguchi-Tanaka et al., 2005; Sun, 2010). In *Arabidopsis*, *GID1* homologs have overlapping but partially specialized functions, supporting the possibility that different *GmGID* members may also have non-redundant roles (Griffiths et al., 2006). Recent soybean evidence further showed that editing a GA receptor gene, *GmGID1-2*, can affect the plant architecture, yield-related traits, seed oil accumulation, and nitrogen fixation, indicating the agronomic relevance of GID-mediated regulation in soybean (Tang et al., 2026). However, as only three representative genes were examined in this study, the GA responsiveness of the whole *GmGID* family requires further validation.

The chloroplast-associated localization of *GmGID5* is noteworthy because canonical GID1 proteins are generally regarded as soluble GA receptors involved in GA perception and DELLA regulation (Ueguchi-Tanaka et al., 2005; Sun, 2010). If confirmed, this localization may indicate functional divergence within the expanded soybean *GID* family. Chloroplasts participate not only in photosynthesis but also in hormone metabolism and retrograde signaling; therefore, *GmGID5* may potentially link GA-related signaling with chloroplast-derived metabolic or stress signals (Rochaix and Ramundo, 2017). Nevertheless, this interpretation remains preliminary as the present localization analysis relied on chloroplast autofluorescence. Future studies using chloroplast-specific markers, stable transformation systems, GA binding assays, and DELLA interaction assays are needed.

The GO enrichment and miRNA–target prediction provided additional hypotheses that *GmGID* genes may be involved in GA signaling, reproductive development, stress responses, and metabolic regulation; however, these predictions require experimental validation. Genome assembly quality should also be considered when interpreting the *GmGID* family expansion. Although telomere-to-telomere (T2T) assembly technologies have greatly improved genome completeness, duplicated and repetitive regions may still affect gene family identification. Future analyses using T2T soybean genomes and pangenome resources may further refine the copy number, chromosomal localization, and evolutionary interpretation of the *GmGID* genes (Yang et al., 2025). Overall, this study provides a useful foundation for future functional characterization of the *GmGID* genes in soybean growth, development, and genetic improvement (Yang et al., 2025).

Overall, this study provides a basis for understanding the evolution, expression divergence, and potential functions of the *GmGID* gene family in soybean. Nevertheless, the GA responses were validated for only three representative genes, subcellular localization was experimentally examined only for *GmGID5*, and the predicted GO/miRNA-related functions require further genetic and biochemical confirmation. Genome assembly quality should also be considered when interpreting the expansion of the *GmGID* family because duplicated and repetitive regions may still affect gene family identification even in the T2T era. Future studies integrating T2T soybean genomes, pangenome resources, CRISPR/Cas-mediated mutagenesis, protein–protein interaction assays, GA sensitivity tests, and physiological analyses will help clarify the copy number, evolutionary history, and biological roles of individual *GmGID* genes in soybean growth and genetic improvement.

.

## Data Availability

The original contributions presented in the study are included in the article/**Supplementary Material**. Further inquiries can be directed to the corresponding authors.

## References

[B1] Anderson N. R. Irizarry M. D. Bloomingdale C. A. Smith D. L. Bradley C. A. Delaney D. P. . (2017). Effect of soybean vein necrosis on yield and seed quality of soybean. Can. J. Plant Pathol. 39, 334–341. doi: 10.1080/07060661.2017.1354333. PMID: 37339054

[B2] Bailey T. L. Johnson J. Grant C. E. Noble W. S. (2015). The MEME suite. Nucleic Acids Res. 43, W39–W49. doi: 10.1093/nar/gkv416. PMID: 25953851 PMC4489269

[B3] Blanco-Touriñán N. Legris M. Minguet E. G. Costigliolo-Rojas C. Nohales M. A. Iniesto E. . (2020). COP1 destabilizes DELLA proteins in Arabidopsis. PNAS 117, 13792–13799. doi: 10.1073/pnas.1907969117. PMID: 32471952 PMC7306988

[B4] Camacho C. Coulouris G. Avagyan V. Ma N. Papadopoulos J. Bealer K. . (2009). BLAST: architecture and applications. BMC Bioinf. 10, 421. doi: 10.1186/1471-2105-10-421. PMID: 20003500 PMC2803857

[B5] Chen C. Chen H. Zhang Y. Thomas H. R. Frank M. H. He Y. . (2020). TBtools: An integrative toolkit developed for interactive analyses of big biological data. Mol. Plant 13, 1194–1202. doi: 10.1016/j.molp.2020.06.009. PMID: 32585190

[B6] Dill A. Jung H.-S. Sun T. (2001). The DELLA motif is essential for gibberellin-induced degradation of RGA. PNAS 98, 14162–14167. doi: 10.1073/pnas.251534098. PMID: 11717468 PMC61185

[B7] Du H. Fang C. Li Y. Kong F. Liu B. (2023). Understandings and future challenges in soybean functional genomics and molecular breeding. J. Integr. Plant Biol. 65, 468–495. doi: 10.1111/jipb.13433. PMID: 36511121

[B8] Duvaud S. Gabella C. Lisacek F. Stockinger H. Ioannidis V. Durinx C. (2021). Expasy, the Swiss Bioinformatics Resource Portal, as designed by its users. Nucleic Acids Res. 49, W216–W227. doi: 10.1093/nar/gkab225. PMID: 33849055 PMC8265094

[B9] El-Gebali S. Mistry J. Bateman A. Eddy S. R. Luciani A. Potter S. C. . (2018). The Pfam protein families database in 2019. Nucleic Acids Res. 47, D427–D432. doi: 10.1093/nar/gky995. PMID: 30357350 PMC6324024

[B10] Fonouni-Farde C. Tan S. Baudin M. Brault M. Wen J. Mysore K. S. . (2016). DELLA-mediated gibberellin signalling regulates Nod factor signalling and rhizobial infection. Nat. Commun. 7, 12636. doi: 10.1038/ncomms12636. PMID: 27586842 PMC5025792

[B11] Gazara R. K. Moharana K. C. Bellieny-Rabelo D. Venancio T. M. (2018). Expansion and diversification of the gibberellin receptor GIBBERELLIN INSENSITIVE DWARF1 (GID1) family in land plants. Plant Mol. Biol. 97, 435–449. doi: 10.1007/s11103-018-0750-9. PMID: 29956113

[B12] Goodstein D. M. Shu S. Howson R. Neupane R. Hayes R. D. Fazo J. . (2011). Phytozome: a comparative platform for green plant genomics. Nucleic Acids Res. 40, D1178–D1186. doi: 10.1093/nar/gkr944. PMID: 22110026 PMC3245001

[B13] Griffiths J. Murase K. Rieu I. Zentella R. Zhang Z.-L. Powers S. J. . (2006). Genetic characterization and functional analysis of the GID1 gibberellin receptors in Arabidopsis. Plant Cell 18, 3399–3414. doi: 10.1105/tpc.106.047415. PMID: 17194763 PMC1785415

[B14] Guo Y. Wu H. Li X. Li Q. Zhao X. Duan X. . (2017). Identification and expression of GRAS family genes in maize (Zea mays L.). PloS One 12, e0185418. doi: 10.1371/journal.pone.0185418. PMID: 28957440 PMC5619761

[B15] Herman E. M. Schmidt M. A. (2016). The potential for engineering enhanced functional-feed soybeans for sustainable aquaculture feed. Front. Plant Sci. 7, 440. doi: 10.3389/fpls.2016.00440. PMID: 27092158 PMC4820450

[B16] Hu B. Jin J. Guo A.-Y. Zhang H. Luo J. Gao G. (2014). GSDS 2.0: an upgraded gene feature visualization server. Bioinformatics 31, 1296–1297. doi: 10.1093/bioinformatics/btu817. PMID: 25504850 PMC4393523

[B17] Hung J.-H. Weng Z. (2016). Sequence alignment and homology search with BLAST and ClustalW. Cold Spring Harbor Protoc. 2016, pdb.prot093088. doi: 10.1101/pdb.prot093088. PMID: 27574197

[B18] Krzywinski M. Schein J. Birol İ. Connors J. Gascoyne R. Horsman D. . (2009). Circos: an information aesthetic for comparative genomics. Genome Res. 19, 1639–1645. doi: 10.1101/gr.092759.109. PMID: 19541911 PMC2752132

[B19] Kumar S. Stecher G. Li M. Knyaz C. Tamura K. (2018). MEGA X: Molecular evolutionary genetics analysis across computing platforms. Mol. Biol. Evol. 35, 1547–1549. doi: 10.1093/molbev/msy096. PMID: 29722887 PMC5967553

[B20] Li W. Wang D. Hong X. Shi J. Hong J. Su S. . (2023). Identification and validation of new MADS-box homologous genes in 3010 rice pan-genome. Plant Cell Rep. 42, 975–988. doi: 10.1007/s00299-023-03006-9. PMID: 37016094

[B21] Li A. Yang W. Li S. Liu D. Guo X. Sun J. . (2013). Molecular characterization of three GIBBERELLIN-INSENSITIVE DWARF1 homologous genes in hexaploid wheat. J. Plant Physiol. 170, 432–443. doi: 10.1016/j.jplph.2012.11.010. PMID: 23261263

[B22] Lohani N. Babaei S. Singh M. B. Bhalla P. L. (2021). Genome-wide in silico identification and comparative analysis of Dof gene family in Brassica napus. Plants 10, 709. doi: 10.3390/plants10040709. PMID: 33916912 PMC8067633

[B23] Mistry J. Chuguransky S. Williams L. Qureshi M. Salazar G. A. Sonnhammer E. L. L. . (2020). Pfam: The protein families database in 2021. Nucleic Acids Res. 49, D412–D419. doi: 10.1093/nar/gkaa913. PMID: 33125078 PMC7779014

[B24] Noor F. (2017). Effects of gibberellic acid (Ga3) on growth and yield parameters of French bean (Phaseolus vulgaris L.). J. Asiatic Soc. Bangladesh Sci. 43 (1), 49–60. doi: 10.3329/JASBS.V43I1.46243. PMID: 40208441

[B25] Ramon U. Weiss D. Illouz‐Eliaz N. (2020). Underground gibberellin activity: differential gibberellin response in tomato shoots and roots. New Phytol. 229, 1196–1200. doi: 10.1111/nph.16876. PMID: 32790883

[B26] Rochaix J.-D. Ramundo S. (2017). Chloroplast signaling and quality control. Essays Biochem. 62, 13–20. doi: 10.1042/ebc20170048. PMID: 29273583

[B27] Sun T. P. (2010). Gibberellin-GID1-DELLA: a pivotal regulatory module for plant growth and development. Plant Physiol. 154, 567–570. doi: 10.1104/pp.110.161554. PMID: 20921186 PMC2949019

[B28] Tang J. Yang S. Li S. Yue X. Jin T. Yang X. . (2026). Editing a gibberellin receptor gene improves yield and nitrogen fixation in soybean. J. Integr. Plant Biol. 68, 75–95. doi: 10.1111/jipb.70026. PMID: 40911442 PMC12782893

[B29] The UniProt Consortium . (2015). UniProt: a hub for protein information. Nucleic Acids Res. 43, D204–D212. doi: 10.1093/nar/gku989. PMID: 25348405 PMC4384041

[B30] To V.-T. Shi Q. Zhang Y. Shi J. Shen C. Zhang D. . (2020). Genome-wide analysis of the GRAS gene family in barley (Hordeum vulgare L.). Genes 11, 553. doi: 10.3390/genes11050553. PMID: 32423019 PMC7290968

[B31] Ueguchi-Tanaka M. Ashikari M. Nakajima M. Itoh H. Katoh E. Kobayashi M. . (2005). GIBBERELLIN INSENSITIVE DWARF1 encodes a soluble receptor for gibberellin. Nature 437, 693–698. doi: 10.1038/nature04028. PMID: 16193045

[B32] Wang H. Jiang H. Xu Y. Wang Y. Zhu L. Yu X. . (2020). Systematic analysis of gibberellin pathway components in Medicago truncatula reveals the potential application of gibberellin in biomass improvement. Int. J. Mol. Sci. 21, 7180. doi: 10.3390/ijms21197180. PMID: 33003317 PMC7582545

[B33] Wang Y. Li J. Paterson A. H. (2013). MCScanX-transposed: detecting transposed gene duplications based on multiple colinearity scans. Bioinformatics 29, 1458–1460. doi: 10.1093/bioinformatics/btt150. PMID: 23539305

[B34] Wang Y. Tang H. DeBarry J. D. Tan X. Li J. Wang X. . (2012). MCScanX: a toolkit for detection and evolutionary analysis of gene synteny and collinearity. Nucleic Acids Res. 40, e49. doi: 10.1093/nar/gkr1293. PMID: 22217600 PMC3326336

[B35] Wang Y. Tang H. Wang X. Sun Y. Joseph P. V. Paterson A. H. (2024). Detection of colinear blocks and synteny and evolutionary analyses based on utilization of MCScanX. Nat. Protoc. 19, 2206–2229. doi: 10.1038/s41596-024-00968-2. PMID: 38491145

[B36] Wu X. Gao T. Shang B. Geng M. Li L. Feng X. . (2024). Effect of ultrasonic treatment on soybean oil: focus on degumming of soybean crude oil and the quality of degummed oil. Eur. J. Lipid Sci. Technol. 126, e202400115. doi: 10.1002/ejlt.202400115. PMID: 41531421

[B37] Wu R. Liu W. Liu K. Liang G. Wang Y. (2023). Genome-wide identification and expression of the GRAS gene family in oat (Avena sativa L.). Agronomy 13, 1807. doi: 10.3390/agronomy13071807. PMID: 30654563

[B38] Xu P. Chen H. Li T. Xu F. Mao Z. Cao X. . (2021). Blue light-dependent interactions of CRY1 with GID1 and DELLA proteins regulate gibberellin signaling and photomorphogenesis in Arabidopsis. Plant Cell 33, 2375–2394. doi: 10.1093/plcell/koab124. PMID: 34046684 PMC8364249

[B39] Yamaguchi I. Nakajima M. Park S.-H. (2016). Trails to the gibberellin receptor, GIBBERELLIN INSENSITIVE DWARF1. Biosci. Biotechnol. Biochem. 80, 1029–1036. doi: 10.1080/09168451.2016.1148575. PMID: 26927225

[B40] Yang Y. Du W. Li Y. Lei J. Pan W. (2025). Recent advances and challenges in de novo genome assembly. Genomics Commun. 2, e014. doi: 10.48130/gcomm-0025-0015

[B41] Yoshida H. Tanimoto E. Hirai T. Miyanoiri Y. Mitani R. Kawamura M. . (2018). Evolution and diversification of the plant gibberellin receptor GID1. Proc. Natl. Acad. Sci. U.S.A. 115, E7844–E7853. doi: 10.1073/pnas.1806040115. PMID: 30068603 PMC6099883

[B42] Yu H. Xia L. Zhu J. Xie X. Wei Y. Li X. . (2025). Genome-wide analysis of the MADS-box gene family in mango and ectopic expression of MiMADS77 in Arabidopsis results in early flowering. Gene 935, 149054. doi: 10.1016/j.gene.2024.149054. PMID: 39490648

[B43] Zhang J. Misra S. Wang H. Feng W. (2016). muBLASTP: database-indexed protein sequence search on multicore CPUs. BMC Bioinf. 17, 443. doi: 10.1186/s12859-016-1302-4. PMID: 27809763 PMC5096327

[B44] Zhang J. Sun A. (2009). Genome-wide comparative analysis of the metalloprotease ftsH gene families between Arabidopsis thaliana and rice. Sheng wu gong cheng xue bao 25, 1402–1408. 19938485

