# Supplementary material for: Genome-wide analysis of the GID gene family in soybean and analysis of expression under gibberellin treatment
Source: Front Plant Sci. 2026 May 20;17:1824314. doi: 10.3389/fpls.2026.1824314 (PMC13230030; doi:10.3389/fpls.2026.1824314)
Supplement: Supplementary file 1 [file Table1.docx]

**Primer sequences**

pCAMBIA1302-GmGID5-GFP-F：catctggcggaggtcagatctATGGCTGGCAGCAACCAA

pCAMBIA1302-GmGID5-GFP-R：gctctgcaggtcgacactagtTTAACAGTCAGAATCAGAATTGACAAAG

GmGID1-qP-F: ACGGTACTTTCAACCGGGAC

GmGID1-qP-R: GCTCACAGGCTTCTCAAGGT

GmGID2-qP-F: AGAAGCGAACCGAATCGGAG

GmGID2-qP-R: GCCAAATGGGTTACAAGCCG

GmGID3-qP-F: GACCTTGAGAAGCCCGTGAA

GmGID3-qP-R: TACAGATACCCACCAGGCGA
